# Event-Driven Observer-Based Smart-Sensors for Output Feedback Control of Linear Systems

**DOI:** 10.3390/s17092028

**Published:** 2017-09-04

**Authors:** Leonardo Acho

**Affiliations:** Department of Mathematics, Universitat Politècnica de Catalunya-BarcelonaTech (EEBE), 08930 Barcelona, Spain; leonardo.acho@upc.edu

**Keywords:** smart sensors, event-driven systems, output feedback control, linear systems

## Abstract

This paper deals with a recent design of event-driven observer-based smart sensors for output feedback control of linear systems. We re-design the triggering mechanism proposed in a previously reported system with the implementation of self-sampling data smart sensors; as a result, we improve its performance. Our approach is theoretically supported by using Lyapunov theory and numerically evidenced by controlling the inverted pendulum on the cart mechanism.

## 1. Introduction

Nowadays, digital computers are often used to control a huge number of continuous-time engineering systems (see, for instance [1,2]). Usually, these control realizations involve utilizing both continuous-time and discrete-time data. However, in the classical point of view, digital controlled systems consist of sampling the system’s response uniformly in time. However, using this strategy scheme for data processing results in a conservative usage of digital resources (see, for instance, [3,4,5,6] and references therein). Because sensors communicate frequently with these digital devices through limited bandwidth lines, especially in networked control systems, communication between sensors and the controller should be as small as possible to increase the performance of the closed-loop system against, for instance, congestion data or packet dropouts [3,6,7,8,9,10,11,12,13], among other benefits [14,15,16].

**Figure 1:**
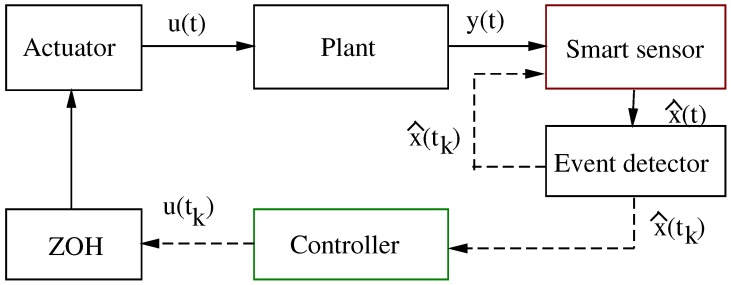
A controlled system, using a smart sensor and based on event-driven observer design: (**1**) solid line for continuous-time data, and (**2**) dotted line for discrete-time signals.

On the one hand, the even-triggered control strategy has been used to relax the homogeneous periodicity of standard sampled-data controlled systems; see, for example, [3,7,16]. This strategy statement also ensures closed-loop system stability on its realization by following well-defined implementation steps. Figure 1 shows a block diagram of its actualization [3,7]. In this scheme, the state of the process, or plant system, is continuously monitored by using an observer algorithm. Then, the smart sensor—based on an event detector signal—generates a sample of the plant state estimation to the controller. Obviously, it is assumed that the sensor has necessary computation capacity for signal processing. The sampled state information is rendered aperiodic because of the event detector system logic. This logic is a well-established rule to ensure closed-loop stability and robustness. For instance, in networked control systems, this strategy design reduces the power requirements, increases its reliability, and simplifies the installation and maintenance of the whole system. Furthermore, it is also robust against packet disorders, the quantization effect, networked-induced delays and their combination. Moreover, and thanks to its self-triggering sampling-rate mechanism, this strategy planning allows the controller to receive less dense signals, hence saving more computational resources and minimizing the bandwidth utilization while still guaranteeing its beneficial closed-loop performance [3,7,17]. Finally, the signal generated by the controller goes to a Zero-Order-Hold device (ZOH), which is immersed in the digital device, to transform discrete-time data into continuous-time information required by the actuator to manipulate the plant.

On the other hand, over the years, several approaches have been developed to deal with non-uniform sampled-data systems, including input-delay approaches [17,18,19,20], impulsive systems formulation [21,22,23], robust analysis techniques [24,25], discrete-time approaches [26,27], clock-time dependent Lyapunov functions [28,29], and Linear Parameter Varying [30,31]. However, in most of these cited references, the triggering mechanism is basically based on the use of the full-state measurement of the plant to be controlled. This action may be problematic because sensors may be sensitive to external noise, and produce the Zeno’s behavior, i.e., requiring an infinite number of samples to be sent in finite-time. Obviously, this situation is not feasible for implementation in practice (see, for instance, [32]). Therefore, event-triggering mechanisms based on observers are desirable [7,13,32]. From the various previously cited approaches proposing an event-triggered control strategy based on observer design, our proposal is based on a modification of the triggering event system stated in [3,7] and is motivated by the one invoked in [6,14] but using the estimation of the states given by the observer mechanism instead of the states of the plant. This proposed strategy greatly improves the controller performance. We support our methodology by using Lyapunov theory. Essentially, the key idea is to stick to a given sampling time only as long as the plant state estimation is outside of a programmed, bounded set around the studied equilibrium point of the closed-loop process. Moreover, an important topic to deal with in self-sampling controller dynamics is the Zeno’s behavior (infinite number of sampling instants in finite ordinary time) which is an undesired phenomenon [3,7,33,34]. In our method, and by employing Lyapunov theory, we give evidence that this behavior is not present in our design. According to numerical experiments applied to the stabilization control of the inverted pendulum on a cart, our approach presents better performance in comparison to a cited case. Finally, it is worth noting that the inverted pendulum has recently been employed to test the performance of novel event-triggered controllers [3,7,14].

The rest of the paper is organized as follows. Section 2 provides the theory on event-triggered controllers based on an observer for output feedback control of linear systems. Then, our main results are presented. Our contribution is theoretically supported by invoking Lyapunov theory; specifically, Input-to-State Stability (ISS) of the proposed closed-loop system is resolved. Section 3 presents the numerical results of our design applied to the stabilization problem of the inverted pendulum in the cart system. We also implement the controller stated in [3,7] for comparison proposes. According to our numerical results, our design shows better performance. Section 4 presents the Results and Discussion of our methodology. Finally, Section 5 presents the main conclusions.

## 2. Event-Triggered System Based on Observers

Considerer the plant model given by [3,7]:

(1) $$\begin{array}{ccc} \dot{x}\left(t\right) & = & Ax(t)+Bu(t) \end{array}$$

(2) $$\begin{array}{ccc} y(t) & = & Cx(t) \end{array}$$

where $x\in R^{n}$ denotes the system state vector, $u\in R^{m}$ represents the control input vector, and $y\in R^{q}$ stands for the plant output vector. The matrices *A*, *B*, and *C* are assumed to be of known appropriate dimensions. Moreover, it is also assumed that (A,B) and (A,C) are controllable and observable systems, respectively. Then—assuming it is a smart sensor—this sensor is able to process information from the plant output y(t) to estimate the state vector of the plant by employing the next well-known Luenberger observer [3,7]:

(3) $$\dot{\hat{x}}\left(t\right)=A\hat{x}\left(t\right)+Bu\left(t\right)+L\left(y\left(t\right)-C\hat{x}\left(t\right)\right)$$

where $\hat{x}\in R^{n}$ is the plant state vector observation, and *L* is the corresponding matrix gain to be designed. On the other hand, the event detector system has to be able to continuously monitor the plant system to generate a firing signal when a programmed event occurs. See Figure 1. Hence, on each firing signal, sampled information is generated to supply discrete-time data to the controller. This is realized by utilizing the plant state vector estimation $\hat{x}\left(t\right)$ and generating its sampled-data, $\hat{x}\left(t_{k}\right)$, obtained at the $t_{k}$ moment of the firing signal. Then, to obtain the next sampling moment, $t_{k+1}$, we propose the following triggering-rule:

(4) $$t_{k+1}=inf\{t>t_{k}\left|\;||e\left(t\right)||>\alpha sat(|\right|\hat{x}\left(t\right)||)+\epsilon_{0}\}\;\forall k\in N,$$

where sat(·) is the saturation function, $e\left(t\right)=\hat{x}\left(t\right)-\hat{x}\left(t_{k}\right)$, and the user given parameter α satisfying 0<α<1 (This restriction is artificially imposed as a kind of *attenuation effect* on the used sampled-data. Obviously, it can be relaxed). $\epsilon_{0}$ is any small positive constant to assure Zeno’s free behavior on the sampling dynamic.

Finally, the event-driven vector control is implemented as follows [3,7]:

(5) $$u\left(t\right)=K\hat{x}\left(t_{k}\right),\;t\in \left[t_{k},t_{k+1}\right)\;k\in N,$$

where *K* is the matrix gain controller of appropriate dimensions. In this way, the controller receives information at $t_{k}$ and keeps its value until the next event activation at $t_{k+1}$. See Figure 1. Next is our main result.

**Theorem** **1.** *The closed-loop system (2), (3), and (5), with the sampling policy stated in (4), is globally uniformly ultimately bounded if* (A+BK) *and* (A−LC)) *are Hurwitz matrices. Moreover, the given sampling policy excludes the Zeno behavior.*

**Proof.**  Actually, our proof follows the same line of thought as used in [3,7]. Then, by defining $\tilde{x}\left(t\right)=x\left(t\right)-\hat{x}\left(t\right)$, and for $t\in [t_{k},t_{k+1})$, we can easily obtain [3,7]:

(6) $$\dot{\tilde{x}}\left(t\right)=\left(A-LC\right)\tilde{x}\left(t\right)$$

Moreover, we have [3,7]:

(7) $$\begin{array}{ccc} \dot{x}\left(t\right) & = & Ax\left(t\right)+BK\hat{x}\left(t_{k}\right) \end{array}$$

(8) $$\begin{array}{cc} \;= & \left(A+BK\right)x\left(t\right)-BK\tilde{x}\left(t\right)-BKe\left(t\right) \end{array}$$

If we define $z\left(t\right)={\left[x^{T}\left(t\right),{\tilde{x}}^{T}\left(t\right)\right]}^{T}$, we have the following closed-loop system representation [3,7]:

(9) $$\dot{z}\left(t\right)=\left[\begin{array}{cc} A+BK & -BK\\ 0 & A-LC \end{array}\right]z\left(t\right)+\left[\begin{array}{c} -BK\\ 0 \end{array}\right]e\left(t\right)=\bar{A}z\left(t\right)+\bar{B}e\left(t\right).$$

Because (A+BK) and (A−LC)) are Hurwitz matrices, there exist positive definite matrices $P=P^{T}>0$, and $Q=Q^{T}>0$ such that:

(10) $${\bar{A}}^{T}P+P\bar{A}+P\bar{B}{\bar{B}}^{T}P=-Q.$$

So, by using the Lyapunov function $V\left(t\right)=z^{T}\left(t\right)Pz\left(t\right)$, we get ([3,7] but the last term:

(11) $$\dot{V}\left(t\right)\leq -\delta V\left(t\right)+\left(\alpha sat(|\right|\hat{x}\left(t\right)||)+\epsilon_{0}),$$

for some positive parameter δ. Finally, and by using the Lemma 4.6 in [35], we can conclude that the closed-loop system (2), (3), and (5), with the sampling policy stated in (4), is globally uniformly ultimately bounded ). Actually, by using this Lemma, we can assure that the cited closed-loop system is *ISS*-stable in the sense of Lyapunov [35]. Furthermore, and because of the globally uniformly ultimately bounded property, the closed-loop system’s trajectory stays around the studied equilibrium point (in our case, the origin of the closed-loop system). Now, to show Zeno’s free behavior of our closed-loop system, we recall the next upper bound of e(t) [3,7]:

(12) $$\left||e\left(t\right)|\right|\leq \frac{\phi (t_{k})}{\left||A|\right|}\left(e^{\left||A||(t-t_{k}\right)}-1\right),$$

for some function $\phi (t_{k})$, see [3,7]. By employing our sampling-rule, we finally obtain

(13) $$\frac{\phi (t_{k})}{\left||A|\right|}\left(e^{\left||A||(t-t_{k}\right)}-1\right)>\epsilon_{0}$$

which means that for any $t_{k+1}$, it should be such that $t_{k+1}-t_{k}>0$. Hence, the Zeno’s phenomenon is missing.

On the other hand, our main vindication for using the saturation function in (4) is to warranty BIBO-stability conceived from (11). At least, let us analyze our closed-loop dynamic at the sampling time moments $t_{k}$. From (11), and by using the Comparison’s Lemma [35], we obtain:

(14) $$\begin{array}{ccc} V(t) & \leq & e^{-\delta t}V\left(0\right)+\int_{o}^{t}e^{-\delta (t-s)}\left(\alpha sat(||x\left(s\right)||\right)+\epsilon_{0}{)}^{2}ds\\ & \leq & e^{-\delta t}V\left(0\right)+{\left(\alpha +\epsilon_{0}\right)}^{2}e^{-\delta t}\int_{0}^{t}e^{\delta s}ds\\ & \leq & e^{-\delta t}V\left(0\right)+\frac{{\left(\alpha +\epsilon_{0}\right)}^{2}}{\delta }\left(1-e^{-\delta t}\right). \end{array}$$

Hence, at sampling time instants, we have:

(15) $${lim}_{k\to \infty }V\left(t_{k}\right)\leq V\left(0\right)+\frac{{\left(\alpha +\epsilon_{0}\right)}^{2}}{\delta },$$

which corresponds to an ultimately bounded sequence. This concludes our proof. ☐

**Remark** **1.** The parameter α used in the policy rule (4) governs the number of sampling-time moments in a certain time-interval.

**Remark** **2.** According to the ISS-theory, the closed-loop system trajectory asymptotically converges to a closed and bounded set in the state-space plot. Although the size of this ultimately bounded set can be straightforwardly estimated from the Lyapunov theory, it is adjustable by manipulating the controller parameters (including the parameter in the event-triggering law) and in trade-off with the number of sampling times. Keeping this set smaller, we require more sampling times. The best option, as in PID controllers, is to tune the controller parameters in line with the experiment in front of many external factors ignored in the control design; for example, delay and bandwidth limitation of the communication channel between the sensor and the controller, etc.

**Remark** **3.** It is worth noting that we are using the Luenberger observer as part of the smart sensor. This is important to highlight because, for instance, in networked control systems, the communication channels, which induce delay signals, introduce noises, and generate packet dropouts, etc., which are located between the controller and the smart sensor, and between the controller and the actuator’s process. Hence, strictly speaking, our implementation is not a state-feedback based on Luenberger observer controller design in the classical sense.

## 3. Numerical Experiments

**Figure 2:**
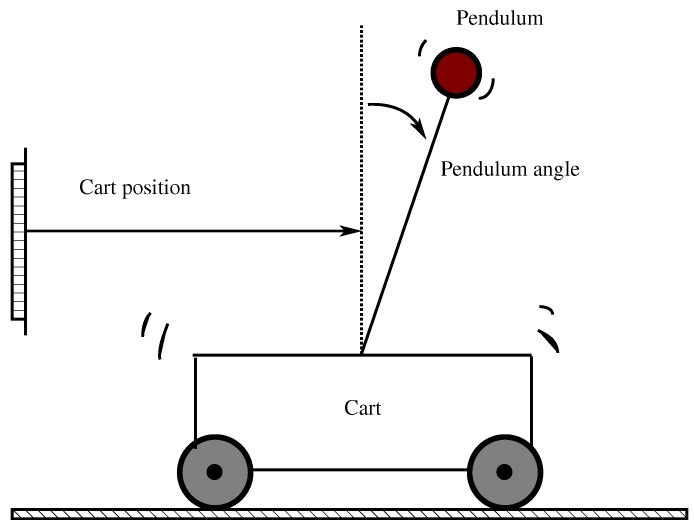
Inverted pendulum on a cart system.

For a numerical performance evaluation of our event-triggered system based on a smart sensor device, consider the inverted pendulum with a cart system (see Figure 2) given by [3,7]:

$$A=\left[\begin{array}{cccc} 0 & 1 & 0 & 0\\ 0 & -0.1789 & 7.7447 & 0\\ 0 & 0 & 0 & 1\\ 0 & -0.5263 & 51.5789 & 0 \end{array}\right]\;B=\left[\begin{array}{c} 0\\ 1.7895\\ 0\\ 5.2632 \end{array}\right]$$

$$C=\left[\begin{array}{cccc} 1 & 0 & 0 & 0\\ 0 & 0 & 1 & 0 \end{array}\right]$$

The above data correspond to a mass cart of M=0.5 kg, pendulum mass m=0.5 kg, pendulum length l=0.3 m, pendulum inertia I=0.006 kg·m${}^{2}$, and cart surface friction b=0.1 N/m/s ([3,7]). Furthermore, consign the next complementary data for the observer part ([3,7])

$$L=\left[\begin{array}{cc} 11.0993 & -0.0991\\ 29.7908 & 2.1306\\ -0.5518 & 11.3189\\ -5.5941 & 63.2481 \end{array}\right]$$

and

$$K=\left[\begin{array}{cccc} 17.0386 & 13.0877 & -50.0520 & -9.8150 \end{array}\right]$$

**Figure 3:**
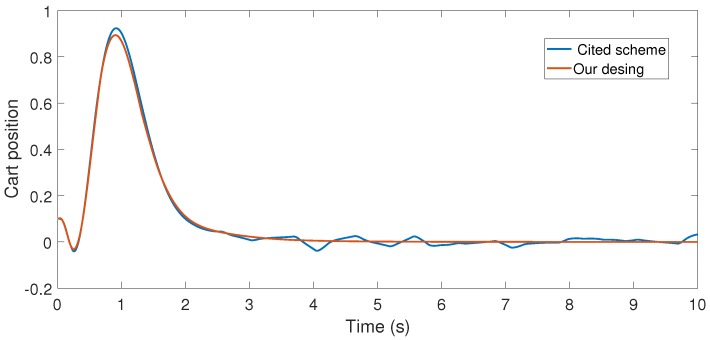
Numerical experiment results: cart position (m).

**Figure 4:**
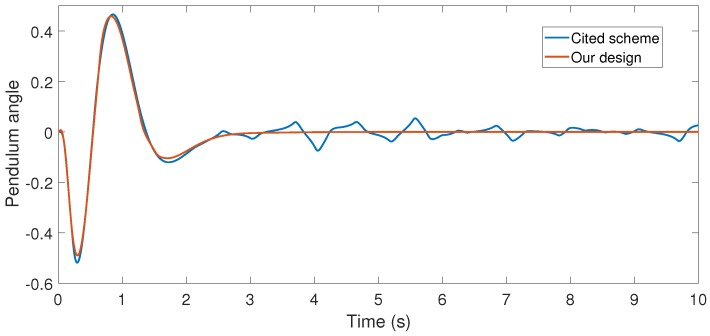
Numerical experiment results: pendulum angle (Rad).

**Figure 5:**
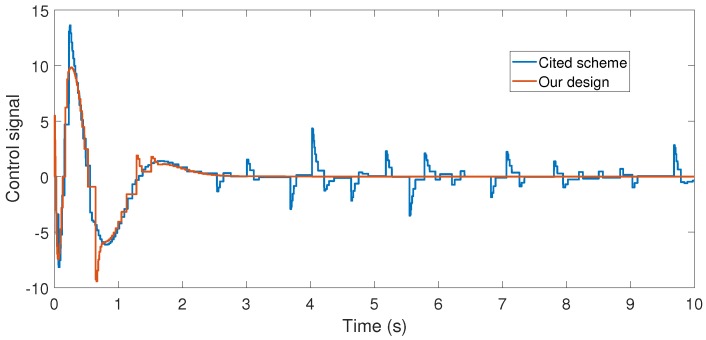
Numerical experiment results: control signal (N).

**Figure 6:**
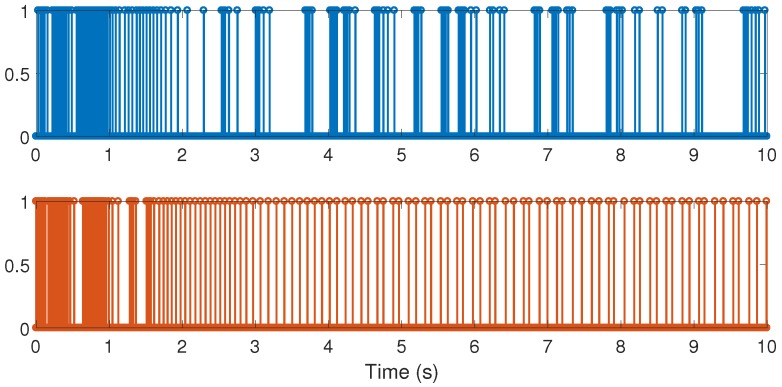
Numerical experiment results: sampling instants; top corresponds to the cited reference case and bottom to our design method.

**Figure 7:**
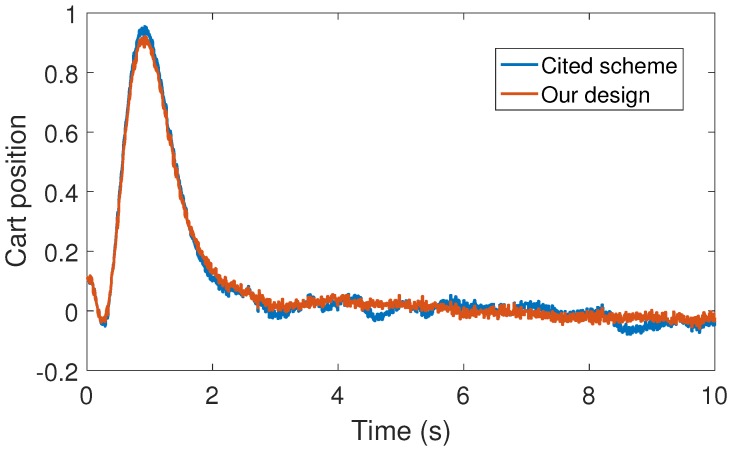
Numerical experiment results(noisy case): cart position (m).

**Figure 8:**
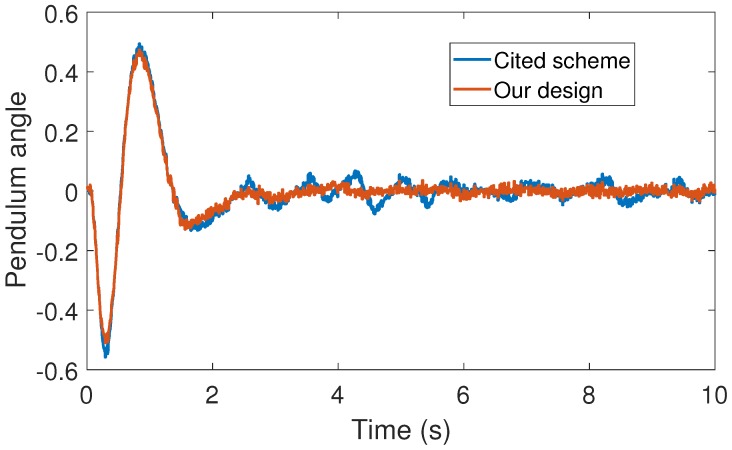
Numerical experiment results (noisy case): pendulum angle (Rad).

**Figure 9:**
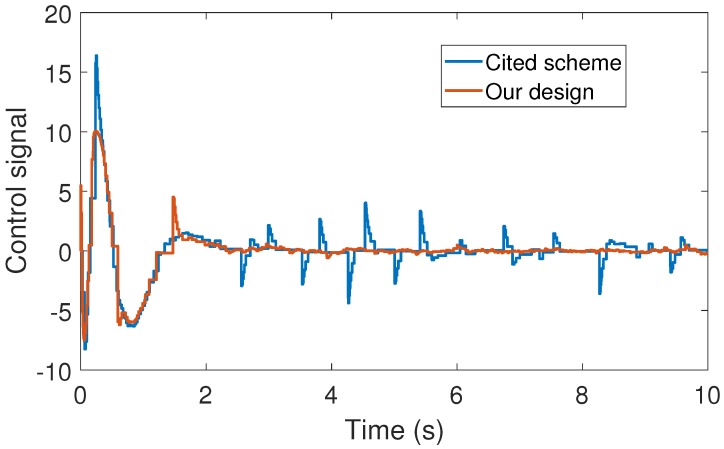
Numerical experiment results(noisy case): control signal (N).

**Figure 10:**
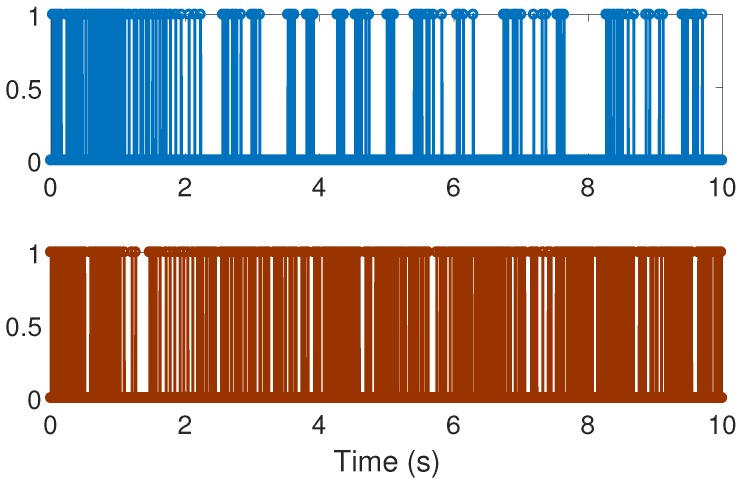
Numerical experiment results (noisy case): sampling instants; top corresponds to the cited reference case and bottom to our design method.

With the above data, the Theorem 1 requirements are satisfied. In our design, we use α=0.2 with plant initial condition ${\left[x,\dot{x},\theta ,\dot{\theta }\right]}^{T}={\left[0.1,0,0,0\right]}^{T}$; where x:=x(t), and θ:=θ(t) are the cart and pendulum position and angle displacement, respectively. The remaining initial conditions were set to zero. For oddity, we employ $\epsilon_{0}=0$. For comparison proposes, we program the event-triggered controller based on observer design stated in [3,7], and we called it the *Cited scheme*. According to the numerical experiments shown in Figure 3 and Figure 4, our design presents better performance. Moreover, Figure 5 shows the corresponding generated control signal; we can again note an improvement in the control energy required to satisfy the regulation control objective of the mechanical system under study. Moreover, Figure 6 shows the triggering moments for each experiment, where we can observe that the Zeno behavior is missing for both cases. Furthermore, by adding white noise to the output plant equation with mean zero and variance 0.0001, the noisy sensor measurements for performance evaluation are shown in Figure 7, Figure 8, Figure 9 and Figure 10. From these figures, we can again note the better performance of our design. Additionally, the noisy case is not studied in the relevant cited references [3,7]. Lastly, from the given numerical results, specifically from Figure 3, Figure 4 and Figure 5, our control design requires less control effort to achieve better results in comparison to the cited design. This may be evidence of the advantages of our approach.

## 4. Results and Discussion

From the experimental point of view, our smart sensor is able to supply non-uniform sampled-data to our control algorithm. These data are supplied according to the closed-loop system dynamics and based on our regulation control objective. Moreover, and again from the experimental results, our design is robust against sensor additive noise, which is a realistic situation in many control system realizations. With respect to the other reported techniques based on plant-state information (see the Introduction section), our design has a filtering property because of the employed observed design, especially for the triggering mechanism. This improves the robustness of our design against unexpected effects such as un-modeled non-linear dynamic terms in the plant, and so on. However, an important scientific point to improve is to extend our main result to the nonlinear case. This is reserved for a future work.

Finally, it is worth noting that the inverted pendulum is a classic problem in dynamics and control theory, widely used as a benchmark for testing new control algorithms (see, for instance, [36]). It is well known that the inverted pendulum is related to rocket or missile guidance. The understanding of similar problems may offer the opportunity to apply our result to other engineering systems, such as the self-balancing personal transporter Segway unit [37], the self-balancing hover-board, the self-balancing unicycle, and the roll stabilization system for monohull ships [38], among others.

## 5. Conclusions

In this paper, we have presented a novel modification to an event-driven observed-based output feedback technique applied to linear systems that, according to numerical experiments, shows better performance. Moreover, the implementation of the proposed event-triggering scheme, which is on the sensor side, only needs to estimate the system’s state at the self-generated sampling times and hence it does not require extra analog hardware to detect the given triggering condition.

To highlight our main contribution with respect to other techniques, the proposed technique is based on the state estimation of the plant, even for the triggering mechanism. As we are using an observer system, it has a filtering property that improves the control performance. Hence, our smart sensor dynamically auto-generates aperiodic sampled-data to our controller with this filtering attribute. This is important, for instance, to help to avoid the undesired Zeno’s phenomenon. Furthermore, our contribution is theoretically supported by using the well known Lyapunov’s theory.

In summary, we have offered a new self-generated sampling-rate smart sensor to a non-uniformly sampled-data control design applied to linear systems. In fact, this topic represents the state-of-the-art in networked-control system applications.

On the other hand, today, remote sensors are commonly used in many applications, for instance, in wind turbines [39]. Hence, an interesting future work on this topic could be the control and health monitoring of wind turbines in Wind Farms by using remote sensing data.

Finally, from the modeling point of view, a sample-and-hold device can be modeled by using a filter dynamic with a “stop-and-go” programming logic, as follows (see details, for instance, [21,40]):

(16) $$\dot{y}\left(t\right)=p\left(t\right)\left[\alpha (-y(t)+u(t))\right];\;x,y,u\in R$$

where α is the time-constant of the filter and p(t) is a non-uniform square signal taking values from the set {0,1}. In this way, when p(t)=0, the solution y(t) in (16) is held (the filter dynamic is stopped) to the last value of u(t) until p(t)=1, where y(t) will follow the signal u(t) (the filter dynamic is released). It is clear that if α is sufficiently large, and the square signal p(t) is too narrow when p(t)=1, then the above dynamic is a kind of ideal sampled-and-hold mechanism. In fact, from the circuit point of view, the real sample-and-hold devices use a capacitor which is charged to the sampled value of the signal u(t), which requires a constant-time for charging (equivalent to our case). We use the above ZOH-model system in our numerical experiment realization.
